# Original dataset used in the article “Does Pokémon Go lead to a more physically active life style?”

**DOI:** 10.1016/j.dib.2018.08.115

**Published:** 2018-08-30

**Authors:** Alessandro Gabbiadini, Christina Sagioglou, Tobias Greitemeyer

**Affiliations:** a University of Milano Bicocca, Italy; b University of Innsbruck, Austria

## Abstract

The data presented in this article are related to the research article entitled “Does Pokémon Go lead to a more physically active life style?” (Gabbiadini et al., in press) [1]. In the study, 981 individuals completed a web survey, in which frequency of Pokémon Go usage, overall physically active behavior, and amount of Pokémon Go related physical activity were measured. Regression analyses revealed that Pokémon Go related physical activity significantly reversed the positive effects of the app on participants’ overall physically active behavior, suggesting that the mere adoption of the app does not reliably change people׳s behavior in general. The increase in physical activity levels is rather explained by the exercise required by the game. The data set is made publicly available to enable critical or extended analyzes.

**Specifications Table**

**Table:** 

Subject area	Social Psychology, Media psychology, Health intervention
More specific subject area	Effect of augmented reality video games on active behavior
Type of data	IBM SPSS data file
How data was acquired	Data collection was performed through Amazon Mechanical Turk (MTurk) by adopting an online web survey. Participation was voluntary and responses were anonymous. No foreseeable risks were involved in participating in the study. Written informed consent was obtained from participants.
Data format	Raw
Experimental factors	N/A
Experimental features	Pokémon Go related physical activity significantly reversed the positive effects of the app on participants’ overall physically active behavior, suggesting that the mere adoption of the app does not reliably change people׳s behavior in general. The increase in physical activity levels is rather explained by the exercise required by the game.
Data source location	N/A
Data accessibility	All the data collected and presented in the study are publicly available on the Open Science Framework web site
	URL: https://osf.io/xy5g6/
	doi:10.17605/OSF.IO/XY5G6

**Value of the data**

• This data show that Pokémon Go app usage is associated with players overall physically active behavior
• This positive effect disappeared when controlling for game-related physical activity
• Pokémon Go users are more active, but only because of the game-required activity

## 1 Data

Raw data associated with this article are publicly available and can be found on the Open Science Framework at https://osf.io/xy5g6/

## 2 Experimental design, materials and methods

The study data was carried out in accordance with the code of ethics of the world medical association (Declaration of Helsinki) for experiments involving humans. Data collection was performed through Amazon Mechanical Turk (MTurk). Participation in the study (see Gabbiadini [1]) was voluntary and all participants were informed that responses were anonymous and no foreseeable risks were involved in participating in the study. Written informed consent was also obtained from participants. Nine-hundred and ninety-nine individuals from the United States (617 females, 382 males; mean age = 32.51 years, *SD* = 10.20) participated in our study and received $0.60. To identify subjects who failed to pay close attention, we included one “catch-trial” in our online survey (i.e., “*please answer 2 to this question*”). Eighteen participants failed the attention check. Thus, the final sample consisted of 981 individuals (610 females, 371 males; mean age = 32.55 years, *SD* = 10.16).

After providing demographic data, participants’ attitudes toward physical activity were assessed by adapting the Attitude Regarding Physical Activities for Health and Fitness Scale (Khanet al. [2]; six items; *α* = 0.68). Sample items were “Participation in physical activities reduces the risk of heart diseases” and “To promote better health conditions, people may take part in the sporting activities”, measured on a scale from 1 = “*completely disagree*” to 7 = “*completely agree*”.

Participants’ overall physically active behavior was assessed by measuring both recency and frequency of their physical activity during the month preceding the study (α = .77). The six items adopted for measuring recency of participants’ physical activity were “When was the last time you had (1) a walk for more than 30 minutes / (2) had a run / (3) had a bike ride to get some exercise?” on a scale anchored with 1 = “*more than one month ago*”, 2 = “*about four weeks ago*”, 3 = “*about three weeks ago*”, 4 = “*about two weeks ago*”, 5 = “*about one week ago*”, 6 = “*during the last week*” and 7 = “*yesterday*”. Frequency of past behavior was measured adopting the following items: “How many times have you had (1) a walk for more than 30 minutes / (2) had a run / (3) had a bike ride to get some exercise during the last month?” (anchored with 1 = “*never*”, 2 = “*two times*”, 3 = “*from three to five times*”, 4 = “*from six to eight times*”, 5 = “*from nine to eleven times*”, 6 = “*from twelve to fourteen times*” and 7 = “*every day*”).

Adopting one item, participants were then asked to report their frequency of Pokémon Go app usage. The item was “How often do you use the new Pokémon Go mobile app on your smartphone?” (on a scale from 1 = *never* to 7 = *very often*).

On an exploratory basis, we also assessed the extent to which people share their achievements on social media by adopting the following item “Do you usually share your Pokémon Go achievements on social networks with other friends in the Pokémon Go network?” (anchored with 1 = “*never”* to 7 = “*very often”*).

Finally, the amount of specific physical activity related to the use of Pokémon Go was assessed adopting three items^2^ (α = .73). The items were “How many times have you walked more than 30 minutes/had a run/had a bike ride with the intent of searching for Pokémon Go during the last month?” anchored with 1 = “*never*”, 2 = “*two times*”, 3 = “*from three to five times*”, 4 = “*from six to eight times*”, 5 = “*from nine to eleven times*”, 6 = “*from twelve to fourteen times*” and 7 = “*every day*”.
