# Supplementary material for: Original dataset used in the article “Does Pokémon Go lead to a more physically active life style?”
Source: Data Brief. 2018 Aug 30;20:732–4. doi: 10.1016/j.dib.2018.08.115 (PMC6129683; doi:10.1016/j.dib.2018.08.115)
Supplement: Supplementary file 1 — Supplementary material. [file mmc1.docx]

**Declaration of interest**

Declarations of interest: none
